# Formation of a Fully Anionic Supported Lipid Bilayer to Model Bacterial Inner Membrane for QCM-D Studies

**DOI:** 10.3390/membranes12060558

**Published:** 2022-05-27

**Authors:** Kathleen W. Swana, Terri A. Camesano, Ramanathan Nagarajan

**Affiliations:** 1 Department of Chemical Engineering, Worcester Polytechnic Institute, Worcester, MA 01609, USA; kathleen.w.swana.civ@army.mil; 2 DEVCOM Soldier Center, Natick, MA 01760, USA

**Keywords:** supported lipid bilayer, anionic lipid bilayer, vesicle to bilayer transition, anionic vesicle, bacterial membrane model, mixed lipid membrane

## Abstract

Supported lipid bilayers (SLBs) on quartz crystals are employed as versatile model systems for studying cell membrane behavior with the use of the highly sensitive technique of quartz crystal microbalance with dissipation monitoring (QCM-D). Since the lipids constituting cell membranes vary from predominantly zwitterionic lipids in mammalian cells to predominantly anionic lipids in the inner membrane of Gram-positive bacteria, the ability to create SLBs of different lipid compositions is essential for representing different cell membranes. While methods to generate stable zwitterionic SLBs and zwitterionic-dominant mixed zwitterionic–anionic SLBs on quartz crystals have been well established, there are no reports of being able to form predominantly or fully anionic SLBs. We describe here a method for forming entirely anionic SLBs by treating the quartz crystal with cationic (3-aminopropyl) trimethoxysilane (APTMS). The formation of the anionic SLB was tracked using QCM-D by monitoring the adsorption of anionic lipid vesicles to a quartz surface and subsequent bilayer formation. Anionic egg L-α-phosphatidylglycerol (PG) vesicles adsorbed on the surface-treated quartz crystal, but did not undergo the vesicle-to-bilayer transition to create an SLB. However, when PG was mixed with 10–40 mole% 1-palmitoyl-2-hydroxy-sn-glycero-3-phospho-(1′-rac-glycerol) (LPG), the mixed vesicles led to the formation of stable SLBs. The dynamics of SLB formation monitored by QCM-D showed that while SLB formation by zwitterionic lipids followed a two-step process of vesicle adsorption followed by the breakdown of the adsorbed vesicles (which in turn is a result of multiple events) to create the SLB, the PG/LPG mixed vesicles ruptured immediately on contacting the quartz surface resulting in a one-step process of SLB formation. The QCM-D data also enabled the quantitative characterization of the SLB by allowing estimation of the lipid surface density as well as the thickness of the hydrophobic region of the SLB. These fully anionic SLBs are valuable model systems to conduct QCM-D studies of the interactions of extraneous substances such as antimicrobial peptides and nanoparticles with Gram-positive bacterial membranes.

## 1. Introduction

**Figure 1:**
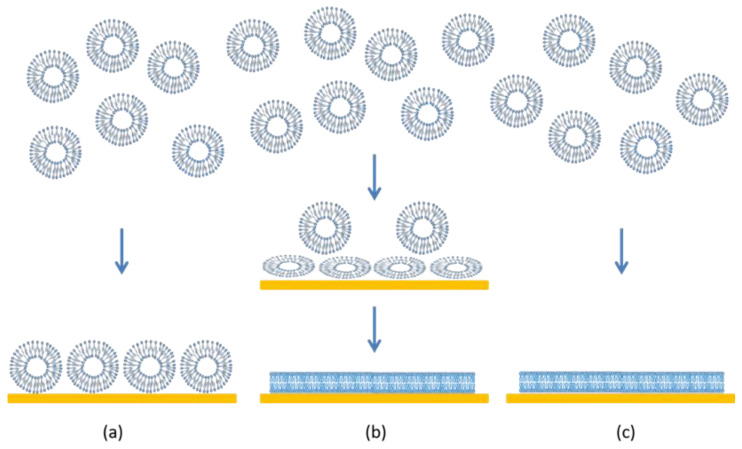
Characteristic scenarios of vesicle–quartz crystal interactions. (**a**) Vesicles adsorb on the surface but retain their shape and structure. No lipid bilayer is formed but a supported vesicle layer results. (**b**) Vesicles adsorb and consequently deform on the surface. In some cases, the vesicles can also fuse. At a critical surface concentration of vesicles, the deformed vesicles rupture to create planar lipid bilayer patches. The edges of the bilayer patch will promote rupturing of surrounding vesicles, resulting in a continuous supported lipid bilayer. (**c**) Vesicles break down immediately on contact with the surface to form planar bilayer patches and the bilayer patches merge to create a continuous planar bilayer.

Supported lipid bilayers (SLBs) are thin planar two-dimensional extended bilayers, that self-assemble near a hydrophilic surface. Due to their planar geometry, the SLBs are amenable to a wide variety of surface sensing techniques and imaging tools such as surface plasmon resonance, quartz crystal microbalance, atomic force microscopy, electrochemical impedance spectroscopy, ellipsometry, infrared spectroscopy and neutron reflectivity [1]. This versatility has contributed to the establishment of SLBs as a widely used model system to study cell membranes. SLBs are commonly prepared by a method pioneered by McConnell et al. [2,3] based on exposing a suspended dispersion of unilamellar lipid vesicles to a suitable surface. When vesicles are exposed to a solid surface, three characteristic outcomes can arise [4] as shown in Figure 1. One is the simple adsorption of the vesicles on the solid substrate, without any significant deformation in the vesicle shape, forming a layer of vesicles. In the second case, the vesicles adsorb on the surface, undergo deformation in shape and in some cases fusion as well, and at a critical concentration of surface coverage, spontaneously rupture to form a SLB. There is also the possibility that some unruptured vesicles remain on the surface even while most of the surface is covered by the planar bilayer. In the third case, the vesicles reaching the surface immediately rupture to form a planar bilayer. Besides these three situations, there is also the less interesting possibility that the vesicles do not interact with the surface at all. If the surface is hydrophobic, there is the likelihood of lipid adsorption as a monolayer, with the polar head groups of the lipids oriented away from the surface.

### 1.1. Quartz Crystal Microbalance with Dissipation Monitoring (QCM-D)

**Figure 2:**
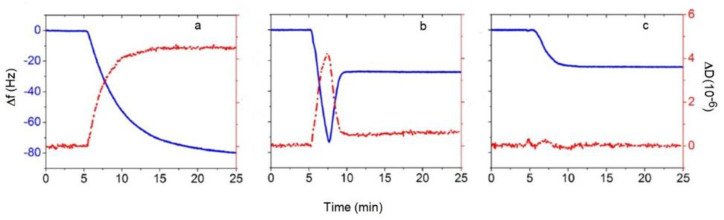
Typical QCM-D responses reflecting the three modes of vesicle–quartz crystal interactions shown in Figure 1. The blue lines are frequency changes with the scale represented on the left axis and the red lines are dissipation changes with the scale represented on the right axis. (**a**) Adsorption of vesicles and formation of a layer of vesicles. (**b**) Adsorption of vesicles followed by their rupture at a critical vesicular coverage and formation of SLB. (**c**) Formation of SLB as soon as the vesicle contacts the substrate. The figures are examples taken from reference [20]. Copyright Jackman et al, Langmuir, 2013.

Quartz crystal microbalance with dissipation monitoring (QCM-D) has become the go-to technique for real-time monitoring of the SLB formation through vesicle fusion/rupture [4,5,6,7,8] as well as for conducting studies on the membrane-mimicking SLB to understand a variety of membrane processes affected by lipid–lipid, lipid–peptide, lipid–protein, lipid–drug and membrane–extraneous nanoparticle interactions [9,10,11,12,13,14,15,16]. The quartz crystal sensor in QCM-D is typically coated with gold or hydrophilic silicon oxide and the hydrophobic gold layer can also be covered with a self-assembled monolayer exposing a hydrophilic moiety. QCM-D provides two important signatures, a change in frequency Δf that can be related to the mass on the crystal surface and a change in dissipation ΔD that can be related to the viscous or viscoelastic properties of the film on the surface. Pioneering studies of SLB formation using QCM-D [4,5,6,7,8,17,18,19,20] have clearly established the unique Δf and ΔD signatures shown in Figure 2, associated with the kinetics of the three types of events arising from vesicle–solid substrate interactions depicted in Figure 1. In case (a), when a stable vesicle layer forms on the quartz crystal without any vesicle deformation and rupture, one obtains a large decrease in the frequency representing the mass of vesicles (lipid as well as entrapped water) and a large dissipation representing a viscoelastic film of vesicles interspersed in the buffer. In case (b), vesicles initially adsorb on the surface indicated by the large decrease in the frequency and the large increase in the dissipation. This is followed by vesicles rupturing, at a critical surface concentration of vesicles, to form the SLB. This is indicated by an increase in frequency associated with the loss of some lipids and water and the decrease in dissipation to low values indicative of a relatively rigid film on the surface. In case (c), on reaching the surface, vesicles immediately rupture to form the SLB. This is shown by the absence of a minimum in the frequency change and the decrease in frequency to a stable value representing the SLB, with little or no change in dissipation throughout the process, indicating formation of a rigid film on the surface.

It should be noted that QCM-D signatures of Δf and ΔD are surface averaged values and do not provide any direct information on the spatial homogeneity of the SLB or the presence of any defects. Nevertheless, the unique QCM-D signatures by themselves have been widely used to monitor the SLB formation in influential studies in the literature cited above and the present work has been conducted following that approach. Other complementary techniques such as atomic force microscopy and fluorescence microscopy can provide morphological information on the SLB, neutron reflectivity may be able to provide lipid compositional features in the case of lipid mixtures and fluorescence recovery after photobleaching (FRAP) measurements may be able to differentiate the lipids in a bilayer from those in a vesicle [1,6,21,22,23,24]. We anticipate using one or more of these techniques in future studies to further characterize the anionic SLB systems we have developed here.

### 1.2. Selecting Lipids for Model Membranes

**Figure 3:**
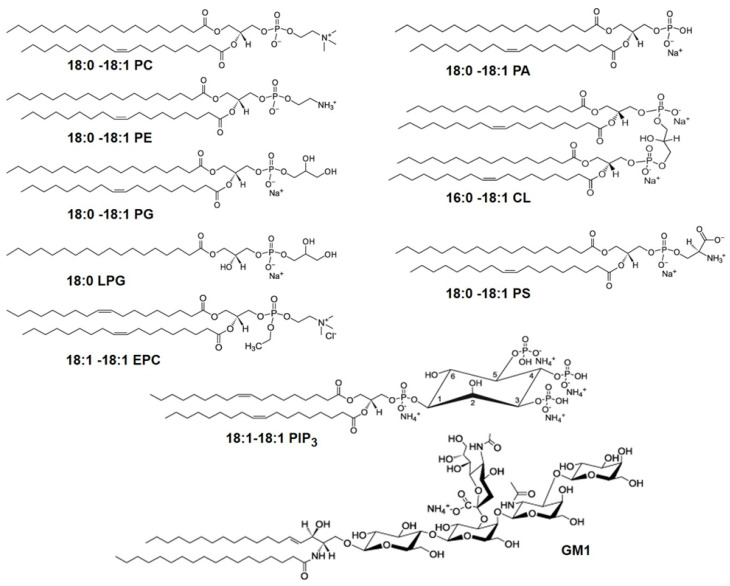
Molecular structures of glycerophospholipids with different head groups referenced in this paper. The numbers designate the chain length and the number of unsaturated bonds in the fatty acid chains of the lipids. The glycerophospholipids with different head groups shown here include: phosphatidylcholine (PC), phosphatidylethanolamine (PE), phosphatidylglycerol (PG), phosphatidylserine (PS), phosphatidic acid (PA), ethyl phosphatidylcholine (EPC), phosphatidylinositol triphosphate (PIP_3_), all with two acyl chains, lysophosphatidylglycerol (LPG) with a single acyl tail and cardiolipin (CL) which has two phosphatidic acid moieties connected via a glycerol backbone forming a dimeric structure, with four acyl chains. Also shown is the glycosphingolipid, monosialoganglioside (GM1) which has a sphingosine and fatty acid chains connected to a branched pentasaccharide head group with a terminal sialyl residue.

Developing a cell membrane-mimicking SLB is a challenging task even if only their lipid components are taken into account. Thousands of different lipid species with hydrophobic acyl chains of varying lengths and levels of saturation connected to various types of hydrophilic head groups are part of the cell membranes, exercising many biological functions [25,26,27]. Figure 3 shows examples of important lipids referenced in this paper. In the erythrocyte plasma membrane, one of the most studied mammalian membranes, the lipid composition includes ∼30 mol% phosphatidyl choline (PC), ∼26 mol% sphingomyelin (SM), ∼27 mol% phosphatidylethanolamine (PE), all zwitterionic, and ∼17 mol% anionic phospholipids, mostly phosphatidyl serine (PS) with some phosphatidyl inositol (PI) and phosphatidic acid (PA) [28]. Concerning the acyl chain composition, acyl chains with 16 and 18 C atoms account for 80 mol% of the total phospholipids of which about 30 mol% exhibit saturated acyl chains, while 43 mol% and 22 mol% are monounsaturated and polyunsaturated, respectively [28].

Table 1 lists the lipid compositions for many bacterial inner membranes [29]. Whereas the plasma membranes of eukaryotic cells are primarily composed of zwitterionic lipids, the bacterial inner membranes of Gram-negative as well as Gram-positive bacteria are typically dominated by anionic lipids with 1–3, phosphatidyl glycerol (PG) and cardiolipin (CL) being the dominant anionic lipids. The fraction of the anionic lipids goes from about 20 mole% in Gram-negative *E. coli* to almost 50 to 90 mole% for many Gram-positive bacteria [29]. Clearly, SLBs with a high content of anionic lipids are needed to represent bacterial inner membranes. A vast majority of the QCM-D studies using SLBs have been conducted primarily with zwitterionic lipids, either as a pure component or as a two or three component mixture. Despite such gross simplicity, they have allowed the QCM-D probing of various molecular interactions between lipids and other molecules relevant to cellular processes.

### 1.3. Past Attempts Incorporating Anionic Lipids in SLBs

SLB models of bacterial inner membranes for QCM-D studies, reported to date in the literature, are few and they are mainly composed of zwitterionic–anionic lipid mixtures with zwitterionic lipids being the major component and containing less than 30 mole percent anionic lipids. Table 2 provides a short summary of these literature studies.

Due to the charges associated with anionic lipids and the anionic quartz crystal (silica) surface, a simple procedure for rapid formation of consistent supported lipid bilayers composed entirely of anionic lipids has proved elusive. Arouri et al. [40] made considerable progress in forming an anionic 1-palmitoyl-2-oleoyl-sn-glycero-3-phosphoglycerol (POPG) supported lipid bilayer on quartz using the Langmuir–Blodgett/Langmuir–Schaefer (LB/LS) technique, but this method is not convenient for use with QCM-D. In this approach, the bilayer would likely need to be formed on a crystal surface outside the QCM-D flow cells, potentially exposing the supported bilayer to air and causing defect formation. Choi and Dimitriadis [41] achieved complete surface coverage of smooth dioleoyl phosphatidylglycerol (DOPG) bilayers on polylysine-coated mica after a 2 h incubation. However, such a method has not yet been successfully implemented in the silica coating of QCM-D sensors. The polylysine coating used in the study by Choi et al. increased the positive charge on the surface, thereby improving its affinity for anionic lipid vesicles. We choose to replicate this concept on our quartz crystal sensors using surface modification with cationic (3-aminopropyl) trimethoxysilane (APTMS) to increase the surface’s affinity for anionic lipid vesicles.

### 1.4. Approach of This Study

Zwitterionic SLBs formed by the vesicle fusion technique have characteristic short formation time (<10 min) and were used in our previous studies to examine the interactions between antimicrobial peptides (AMPs) and model membranes [13,14,15]. To facilitate future experiments to monitor antimicrobial peptide (AMP) interactions with bacterial inner membrane models, we seek to create anionic SLBs within the span of several minutes utilizing the vesicle fusion technique. The role of divalent cations in promoting mixed zwitterionic–anionic lipid vesicles to form SLBs [18,30,32,39], the ability of AOT bilayers to be formed at a low pH when the quartz surface has a positive charge [37], the ability of the zwitterionic–cationic lipid vesicles to form an SLB on the quartz crystal [19] and the formation of an anionic SLB on a cationic polymer polylysine-coated mica [41] all clearly suggest the importance of electrostatic interactions between the vesicles and the substrate in promoting the SLB formation. Guided by this understanding, in this study, we report a simple protocol for forming supported anionic lipid membranes on silica within ~5 min. To enable this, the quartz crystal was functionalized with (3-aminopropyl) trimethoxy silane (APTMS) to make the surface cationic and encourage attachment of anionic lipids. PG was chosen as the anionic lipid for forming the SLB since it is the predominant anionic lipid in bacterial inner membranes. We studied bilayer formation capabilities of various compositions of egg L-α-phosphatidylglycerol (PG) and 1-palmitoyl-2-hydroxy-sn-glycero-3-phospho-(1′-rac-glycerol) (LPG), which have two or one hydrophobic acyl chains, respectively. LPG with a single chain will form spherical micellar aggregates as predicted by theories of molecular packing [42] and confirmed by experiments [43]. We anticipate that the addition of LPG to PG will allow the manipulation of the vesicle size and membrane curvature effects as well as the dynamics of lipid movement involved in the SLB formation process. We report on the kinetics of SLB formation, represented in terms of the QCM-D signatures, since this has been used as an established method to monitor the successful formation of SLBs via vesicle fusion.

## 2. Materials and Methods

### 2.1. Lipids

Egg L-α-phosphatidylglycerol (Egg PG) and 16:0 1-palmitoyl-2-hydroxy-*sn*-glycero-3-phospho-(1′-*rac*-glycerol) (LPG) were purchased from Avanti Polar Lipids (Alabaster, AL, USA). Based on the supplier information, Egg PG has a mixture of acyl chains, with 32.9% of 16:0, 12.2% of 18:0, 30.2% of 18:1 and 18.7% of 18:2. PG and LPG were stored in chloroform at −20 °C. Vesicle solutions containing PG and LPG lipids were suspended in 4-(2-hydroxyethyl)piperazine-1-ethanesulfonic acid (HEPES) buffer (10 mM HEPES, 150 mM NaCl, 2 mM NaN_3_, pH 7.4) with the addition of 2 mM CaCl_2_. The same buffers were used for the initial buffer rinse (before lipid vesicle flow), the vesicle solution and final buffer rinse, in all QCM-D experiments.

### 2.2. Lipid Vesicle Formation

Five anionic lipid membrane compositions were considered in this study to examine the effect of increasing LPG compositions: PG, 9:1 PG/LPG (molar ratio), 8:2 PG/LPG, 7:3 PG/LPG and 6:4 PG/LPG (also referred to as 0%, 10%, 20%, 30% and 40% LPG, respectively). The lipids were dried in the appropriate proportions to remove the chloroform or ethanol solvents and placed in a vacuum desiccator overnight. The dried lipids were suspended in the HEPES buffer with CaCl_2_ to bring the total lipid concentration to 2.5 mg/mL. The lipid solution was mixed well and went through 5 freeze–thaw cycles. The lipids were then sonicated with an ultrasonic dismembrator (Model 150T, Fisher Scientific, Waltham, MA, USA) in pulsed mode for 30 min in a 70 °C water bath. A 30% duty cycle (3 s sonication followed by a 7 s pause) with an amplitude of 60 was used. The vesicles were then centrifuged for 10 min at 16,000× *g* to remove probe particles and large lipid aggregates. The supernatant was removed and stored under nitrogen at 7 °C and diluted to a concentration of 0.1 mg/mL before each experiment.

Dynamic light scattering (Zetasizer Nano ZS, Malvern, Worcestershire, UK) (DLS) was used to measure vesicle size in solution and monitor vesicle stability over time. DLS measures the Brownian motion of the particles in a sample, from which the particle size can be derived. Small particles are known to move quickly in a liquid, while large particles move slowly. The intensity distributions of the suspended lipid vesicle samples were measured and used to generate size distributions of the vesicles by volume.

### 2.3. Surface Treatment of Quartz Crystal

All anionic lipid membranes (PG and PG/LPG) were formed on silica QCM-D sensor crystal surfaces treated with (3-aminopropyl)trimethoxysilane (APTMS). APTMS was used to make the sensor surface cationic to facilitate the attachment of anionic lipid vesicles. Before each experiment, the sensors were rinsed with 2% sodium dodecyl sulfate (SDS) and DI water, dried with nitrogen gas and etched with an SPI Plasma Prep II Plasma Etcher (SPI Supplies, West Chester, PA, USA). To generate the APTMS coating, the sensors were immersed in ethanol for 5 min, methanol for 5 min and a 30 vol% APTMS/methanol mixture for 20 min. After rinsing with methanol and drying with nitrogen gas, the crystals were ready for use in QCM-D experiments. At the end of each experiment, the crystals were rinsed with 2% SDS, DI water and methanol and dried with nitrogen gas. While the above protocol was consistently followed for the APTMS functionalization of the quartz surface, drawing upon earlier experience in our laboratory (unpublished) in treating various hydrophilic surfaces such as glass, silica and cellulose with APTMS, no quantitative characterization of the APTMS attachment to the surface was carried out.

### 2.4. QCM-D Experiments of SLB Formation

The Q-Sense E4 system (Biolin Scientific, Gothenburg, Sweden) was used to monitor the attachment of lipid vesicles to a sensor surface and the subsequent bilayer formation. The quartz crystal with APTMS treatment was taken as the reference system on which further mass changes due to vesicle adsorption and SLB formation were monitored through measurements of the two characteristic QCM-D signatures.

To form SLBs, a baseline was first established in the frequency and dissipation measurements by flowing the buffer that was also used to prepare the lipid vesicle solution. The lipid vesicle solutions were then injected into the QCM-D chambers at 0.15 mL/min and shifts in f and D were measured as the vesicles were given ample time (up to 40 min, requiring 6 mL lipid vesicle solution) to attach to the sensor surface and potentially adsorb and/or form bilayers. Finally, the sensor surface was again rinsed with an injection of the original buffer to remove any weakly attached particles from the surface. All experiments were performed at 23 °C. The changes in frequency and dissipation measured refer to the difference from the initial state of APTMS-treated quartz crystal in contact with the buffer and the final state after vesicle flow followed by the flow of the same buffer. Therefore, the measured changes directly account for the consequences of the interaction of the lipid vesicles with the APTMS-treated quartz surface. Since it is possible to measure Δf and ΔD not only at the fundamental resonant frequency of the quartz crystal, but also in its harmonics, in this study, the 3rd through 11th overtones, or harmonics, were measured and are reported here.

Methods to relate the measured frequency and dissipation changes to changes in mass and in the viscoelastic properties of the material mass on the crystal surface have been described in detail in the literature [44] and a brief summary is provided in Appendix A. It can be noted that if a rigid film of areal mass Δm (mass per unit area) is deposited on the crystal surface, it gives rise to a change in frequency Δf, given by the Sauerbrey equation, as Δm = −C Δf, where C is a constant. If the change in dissipation ΔD is small (less than 1 × 10^−6^), then the deposited film can be considered rigid and the Sauerbrey equation is applicable. If ΔD is much larger, the film is viscoelastic and the change in mass Δm will need to be estimated by considering both Δf and ΔD as shown in Appendix A.

## 3. Results and Discussion

### 3.1. Vesicle Size and Stability

**Figure 4:**
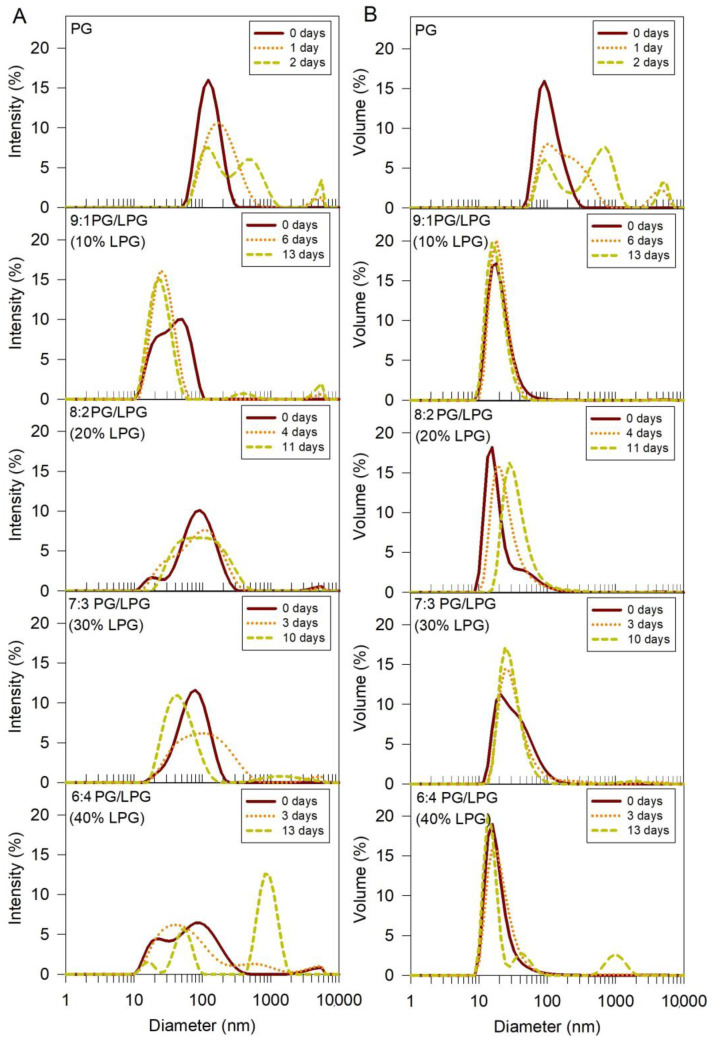
Size distribution of PG and PG/LPG vesicles in order of increasing LPG percentage. The intensity (**A**) and volume (**B**) size distributions were determined using DLS. Size distributions at three time points are presented for each vesicle type to observe the relative stability of the vesicles against aggregation over time.

DLS was used to measure vesicle sizes immediately following the sonication and centrifugation steps of the vesicle formation procedure (t = day 0). Figure 4 shows the size distributions of each experimental vesicle composition by intensity (Figure 4A) and volume (Figure 4B). Since intensity (I) is what is actually measured, it is the direct basis of the size distribution generated by DLS. From the measured intensity distribution, one can theoretically calculate other distributions such as that based on volume (V) or number (*N*). If the solution contains *N_i_* spherical particles of diameter *d_i_*, the various distributions can be represented as

(1) $$\%I\;=100\frac{N_{i}d_{i}^{6}}{\sum N_{i}d_{i}^{6}},\;\%V\;=100\frac{N_{i}d_{i}^{3}}{\sum N_{i}d_{i}^{3}},\;\;\%N=100\frac{N_{i}}{\sum N_{i}}.$$

While the intensity distribution is actually measured, the volume and number distributions are only the calculated values, requiring some assumptions to be made such as the particles being spherical.

The %Intensity distribution emphasizes larger species more than the %Volume or %Number distributions. If the presence or absence of aggregates is of interest, the %Intensity distribution will clearly reveal the presence of the larger species. Additionally, if a small change in size of the particle needs to be monitored, the %Intensity will reveal this better than the other distributions. The %Volume distribution is generally used if the size of the most prominent species (i.e., the size that has the highest concentration in the distribution) is of interest. The %Number distribution will provide the numbers of particles, of varying sizes, if that is of interest. Volume distributions are included in Figure 4B for qualitative assessment of the relative abundance of specific vesicle sizes. It is important to note that since larger particles scatter more light than smaller particles, the area of the intensity peak for larger particles in the size distribution will be greater than the area for smaller particles if the numbers of small and large particles in the solution are the same. The same is true for the volume measurements, since the volume of larger particles is greater than the volume of smaller particles. Therefore, these graphs cannot be used to directly infer the number of particles of each size present in the solution. The relative uniformity and stability of each vesicle type can be inferred from the size distribution plots, however.

DLS size distributions (both intensity and volume) for the freshly prepared vesicles show that the PG vesicles were larger than vesicles containing a mixture of PG and LPG, when formed under the same conditions. The mixed PG/LPG vesicles were less than half the size of PG vesicles containing 0% LPG and were similar in size to PC vesicles (~37 nm) used in our previous studies of SLB formation with zwitterionic Egg PC lipids [14,15]. Vesicle sizes showed some variations depending on the amount of LPG incorporated, but these variations were smaller in comparison to the variation against the PG vesicles.

Stability of the anionic vesicles was also assessed from DLS measurements. Vesicle size distributions were measured at various time points to monitor the aggregation and stability of the vesicles over time (Figure 4). Shifting of the intensity peak towards larger diameter values indicated that some of the particles in the PG vesicle solution increased in size, or aggregated, within 1 day. The presence of a second and third peak after 2 days revealed further growth of particle size, or aggregation. The PG vesicle solutions were therefore sonicated and centrifuged before each QCM-D experiment to ensure that the vesicle sizes were consistent between QCM-D trials. The mixed PG/LPG lipid vesicles were more stable over time (for figure clarity, all time points are not shown). The intensity plots show that vesicles containing 10–20% LPG remained stable for 4–6 days, while vesicles with 30–40% LPG began to aggregate after 3 days. Although the addition of LPG to PG vesicles seems to improve vesicle stability against aggregation, increasing the concentration of LPG to 40% and above appears to promote vesicle instability. Depending on the composition, the PG/LPG vesicles used in these experiments were stored under nitrogen at 7 °C for at most 2–4 days.

The single acyl tail containing LPG would aggregate into micelle structures whereas the double acyl chain containing PG would aggregate into a vesicular structure [42,43]. The single acyl chain LPG is also expected to have increased dynamics of movement between the solvent and vesicle structure, because of the lower hydrophobicity of the single-chain LPG compared to the double-chain PG.

### 3.2. Anionic SLB Formation

**Figure 5:**
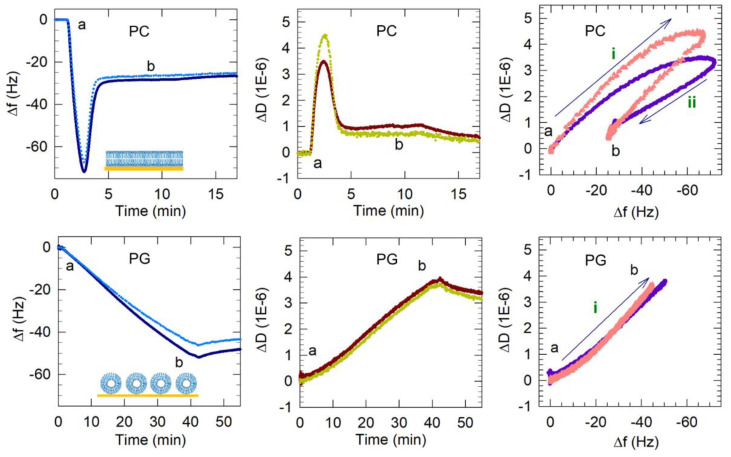
Representative QCM-D results showing the flow of PC vesicles over silica (non-coated) and PG and PG/LPG vesicles over APTMS-coated silica. Time points marking the start and end (a and b) of vesicle flow are represented in the frequency and dissipation shifts measured over time and also in the Δf–ΔD plots. Only the 3rd and 11th harmonics are shown for clarity. Based on these QCM-D results, the final membrane configurations (complete bilayer, incomplete bilayer or vesicle layer) are represented by the graphics in the Δf–time plots. The frequency axes in the Δf–ΔD plots are reversed so that progression of points to the right represents mass increase. The progression to the top indicates increasing viscoelasticity. The arrows (labeled i and ii) show the progression of data points over time and the sequence of different processes occurring on the sensor surface. For PG, only the process i is observed while, for PC, two processes are seen. The data shown for PC bilayer formation were adapted from previous publications from this lab [14,15]. Copyright Wang et al, Colloids Surf. B Biointerfaces, 2014; Wang et al, Biophys.Chem., 2015.

From previous studies on supported PC lipid bilayers formed on bare (unfunctionalized) silica surfaces, vesicle adsorption followed by vesicles rupturing beyond a critical surface concentration of vesicles have been identified as the two principal steps leading to supported lipid membrane formation using the vesicle fusion method [4,5,6,7,8,17,18,19,20]. The frequency and dissipation shifts resulting from typical PC bilayer formation on silica [14,15] are presented in Figure 5. The QCM-D measurements show that an initial frequency decrease of −72 Hz was observed as PC vesicles adsorbed to the sensor surface. During the same time, the dissipation increases with ΔD peaking at a value of 3.5 × 10^−6^ reflecting the hydrated and “soft” vesicles adsorbed. This critical concentration of vesicles is reached within 2 to 3 min of the initiation of the adsorption. The adsorbed vesicles then rupture, releasing water mass as well as lipids. Correspondingly the frequency increased from its minimum observed at the critical vesicle concentration and stabilized around −28 Hz. At the same time, the dissipation decreased, with ΔD stabilizing at a value of 0.6 × 10^−6^ reflecting the formation of a relatively rigid lipid bilayer. A final buffer rinse to remove any weakly attached particles from the membrane brought the frequency to −26 Hz (final Δf). This entire process of the bilayer formation occurs within about 5 min. Since these frequency and dissipation responses are consistently characteristic of PC bilayer formation, we expect the f and D responses associated with anionic bilayer formation to result in similar final Δf and ΔD values (Δf ~−26 Hz and ΔD < 1 × 10^−6^).

#### 3.2.1. PG Vesicles form a Vesicle Layer and Not a Bilayer on APTMS-Coated Silica

The frequency and dissipation shifts resulting from the interactions of the PG vesicles with the quartz crystal are also presented in Figure 5. The Δf and ΔD plots show that Egg PG vesicles did not undergo the same two-step process of vesicle adsorption followed by vesicles rupturing observed for Egg PC vesicles. Instead, the frequency shift decreased to ~−53 Hz and remained near that value, revealing PG vesicles adsorbed on the APTMS-coated surface during 40 min of vesicle flow and remaining stabilized as a supported vesicle layer (SVL) during and after the final buffer rinse. There was no minimum in the frequency change and no subsequent variation as well. At the same time, the dissipation continued to increase, with ΔD reaching a value of 3.8 × 10^−6^. Further, the process of frequency decrease was much slower, almost 40 min, compared to the 2 to 3 min it took for the critical vesicle concentration to be reached for PC vesicles. The final PG layer produced after 40 min of flow resulted in a frequency shift of −52 Hz in the 3rd harmonic (all following reported Δf and ΔD values were extracted from the 3rd harmonic), which was nearly twice as large as the final frequency shift associated with PC bilayer formation (−26 Hz). The large ΔD increase (3.8 × 10^−6^) suggested that the resulting PG film contained more entrapped water or disordered molecules than the rigid PC bilayer. A final dissipation shift greater than 1 × 10^−6^ indicates the presence of a non-rigid viscoelastic film. Therefore, comparing the QCM-D signatures of PG against those for PC, we conclude that a PG bilayer was not formed in this case, even though the surface had been treated to become cationic.

It should be noted that the 40 min period allowed for injection of the PG vesicle solution should have provided adequate time for the vesicles to saturate the sensor surface. As shown in Figure 5, PC bilayer formation on silica requires ~5 min to reach the minimum frequency before adsorbed vesicles may rupture into a bilayer. In preliminary experiments involving PG vesicle flow over the QCM-D sensor (data not shown), frequency measurements did not stabilize even after reaching −75 Hz, which is typically the minimum critical frequency reached before PC vesicles will rupture into a bilayer. This continuing mass addition may have corresponded with the formation of more than one layer of vesicles or the adsorption of vesicle aggregates. Since the formation of a PG bilayer seemed unlikely to occur under these conditions, PG vesicle flow was stopped at 40 min in these experiments. The frequency overtones also separated from each other during mass attachment, as would be expected with hydrated films that are not uniform throughout their thickness (e.g., vesicle layers). The final dissipation value was large (3.8 × 10^−6^), indicating that the film was more hydrated than a stable PC bilayer.

#### 3.2.2. LPG Addition Promotes Bilayer Formation

**Figure 6:**
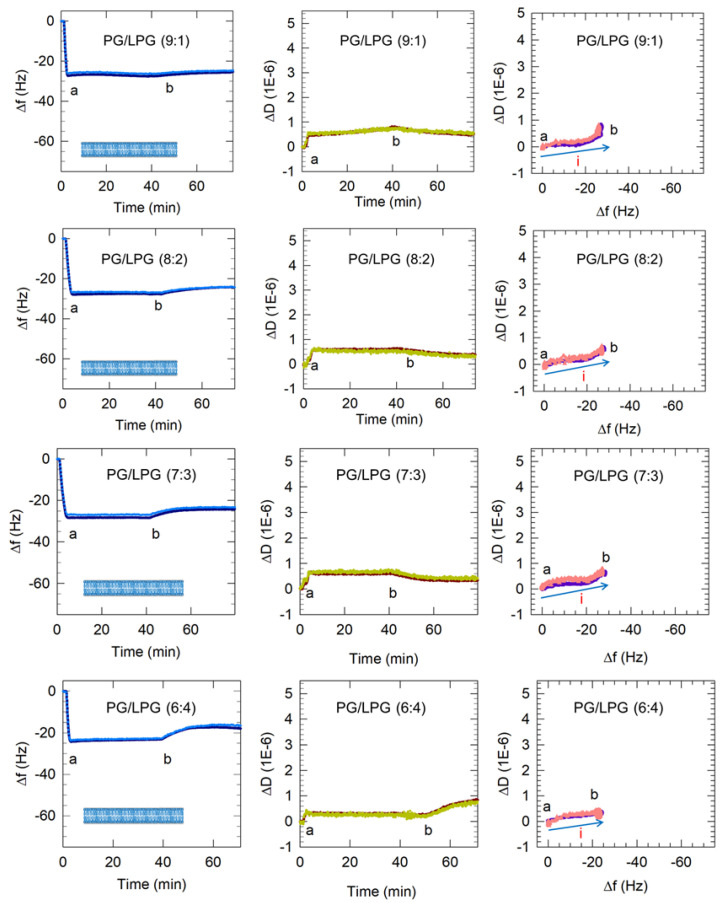
Representative QCM-D results showing the flow of PC vesicles over silica and PG and PG/LPG vesicles over APTMS-coated silica. Time points marking the start and end of vesicle flow, denoted as a and b, are shown in all the plots. Only the 3rd and 11th harmonics are shown for clarity. Based on these QCM-D results, the final membrane configurations for each lipid composition at point b are represented by the graphics in the Δf vs. time plots. In the ΔD vs. Δf plots, the progression of points to the right represents mass increase and the progression to the top indicates increasing viscoelasticity. The blue arrows (labeled i) show the progression of data points over time and the single type of process occurring on the sensor surface. The final buffer rinse following point b indicates some removal of lipid, with the change being significant for the 6:4 PG/LPG system (see discussion below).

The frequency and dissipation shifts resulting from the interactions of the mixed PG/LPG vesicles with the APTMS-coated quartz crystal are presented in Figure 6. A comparison of the initial frequency slopes (from point a) for various vesicle types is shown in Table 3. The slope values were calculated from the time after vesicle flow began to the time when the minimum in the frequency is reached. A greater negative slope suggests more rapid vesicle attachment to the sensor surface. All anionic vesicles in this study exhibited slower surface adsorption than zwitterionic PC vesicles while PG/LPG vesicles adsorbed on the APTMS-coated silica more rapidly than PG vesicles.

Vesicles containing 10–30% LPG formed a stable bilayer within approximately 5 min, which is similar to the PC bilayer formation process. In the case of zwitterionic Egg PC vesicles, the initial vesicle attachment on the quartz crystal resulted in a frequency change of at least −70 Hz shift, indicating the critical vesicle concentration at which vesicle rupture is initiated. The vesicle rupture causes the water from the vesicles’ interior to be released as well as possibly some lipid molecules and this mass loss results in the final frequency change of about −26 Hz, corresponding to the presence of a stable bilayer. In marked contrast, the frequency change resulting from 10–30% LPG vesicles contacting the quartz surface was between −26 and −28 Hz (starting from point a) and there was no subsequent increase in the frequency, implying that no mass loss occurred after the vesicles contacted the substrate. This would confirm that when vesicles reached the quartz surface they instantaneously rupture, releasing all water from the vesicle interior and forming bilayer patches, and do not stay as vesicles at any time following contact with the substrate. Before the final buffer rinse (point b), the membranes containing 10–30% LPG/PG stabilized between Δf = −26 Hz and −29 Hz and ΔD < 1 × 10^−6^. These values are near the Δf and ΔD shifts measured for PC lipid membranes, indicating the successful formation of PG/LPG bilayers, rather than vesicle layers. The final Δf value associated with 10–30% LPG/PG lipid membrane formation revealed slightly more mass on the surface of the sensor than with PC bilayers, which may be a result of the difference in molecular weight between the lipids used.

#### 3.2.3. Increasing LPG to 40% Promotes Lipid Removal from the Membrane

Vesicles containing 40% LPG when contacted with the quartz crystal surface resulted in a final Δf value of −23 Hz (Figure 6). This is somewhat smaller than the values of 26 to 29 Hz observed for lower LPG compositions of the PG/LPG mixture. The buffer rinse at point b in the 40% LPG system brought about an unexpected increase in dissipation (to approximately 0.8 × 10^−6^) at the same time as the frequency increased. This would suggest that the bilayer was losing mass while gaining viscoelasticity. This phenomenon was observed consistently in our experiments and may be explained by single lipids being removed through the buffer rinse. The loss in lipid mass would cause the frequency to increase and the rise in dissipation may be a result of large defects in the membrane after some lipid removal. The final frequency change value of −18 Hz corresponding to a stabilized 40% LPG bilayer is significantly lower than the value of −26 Hz for a stabilized PC bilayer. Even after accounting for the differences in lipid masses (as LPG exhibits a lower molecular mass than PC), clearly the average area per lipid on the bilayer is much larger in this case (as discussed below in Section 3.4). This appreciable low stable value of −18 Hz must therefore be due to bilayer patches not being continuous and not covering the entire quartz crustal surface. Further experimentation using atomic force microscopy (AFM) should be performed to confirm this implication from the QCM-D data and assess the consistency of this membrane. Evidence that the bilayer is still relatively rigid despite the vacancies comes from the uniform overtones (existence of overtone separations in frequency change as well as dissipation is a characteristic of viscoelastic films) and that the dissipation still remained below 1 × 10^−6^.

### 3.3. Dynamics of Vesicle Adsorption and Bilayer Formation

Plots of ΔD vs. Δf in Figure 5 and Figure 6 reveal changes in the dynamic processes that occur on the sensor surface and highlight differences between anionic bilayer formation and SLB formation from the zwitterionic PC. The points in these plots represent Δf and ΔD measurements taken at evenly spaced 0.7 s time intervals and therefore reveal the rate of each process. Processes occurring on the sensor surface, such as vesicle adsorption or rupture, are represented by the direction of the progression of data points over time (labeled with arrows i and ii). It is important to note that the *x*-axis scale in these plots is reversed so that progression of points to the right represents a mass increase and the progression of points to the top represent increase in viscoelasticity. Vesicle adsorption adds mass to the surface, leading to a decrease in frequency and the trapped water between vesicles makes the film viscoelastic, causing an increase in dissipation (or progression in the north-east direction in ΔD vs. Δf plots). Vesicle rupture is accompanied by water mass loss from the adsorbed film, resulting in increasing frequency, and, because the film becomes more rigid, decreasing dissipation (or progression in the south-west direction). These processes can be followed in the ΔD vs. Δf plot for PC bilayer formation in Figure 5, consisting of two clear stages, which represent vesicle adsorption (i) and rupture (ii).

In contrast, PG vesicle adsorption to the surface occurred in a single-stage process, namely just vesicle adsorption (process i), and is not followed by any vesicle rupture. For mixed LPG/PG vesicles, only a single-stage process is observed (Figure 6). However, this process is represented by the line going mainly east and not north-east as in the case of the PC bilayer. The pathway to the east indicates an increase in mass occurring without any accompanying change in the dissipation. This clearly implies that when vesicles reach the crystal surface, they adsorb not as vesicles but directly as patches of bilayer.

### 3.4. Estimation of SLB Molecular Packing Characteristics

As mentioned before, the QCM-D signatures of Δf and ΔD are average values for the film adsorbed on the crystal surface and therefore can be used to determine the film characteristics on average. Using the frequency changes that were observed in Figure 5 and Figure 6, we have estimated the area per lipid (a_L_) in each bilayer and the thickness of the bilayer’s hydrophobic region (h_L_). The difference in molecular weights between LPG, PC and PG are taken into account in arriving at the estimates of bilayer geometric characteristics. LPG, Egg PC and Egg PG have increasing average molecular weights of 506 g/mol, 770 g/mol, 782 g/mol, respectively. Using the 9:1 PG/LPG system as an example, the frequency change (Δf) measured for the stabilized membrane was −25 Hz. Using the expression Δm = −C Δf, (with C = 17.8 ng/cm^2^/Hz for a crystal with a natural frequency of 5 MHz), the areal mass of the film on the quartz crystal was calculated to be 445 ng/cm^2^. If we assume that the anionic bilayer has hydration behavior on the silica surface similar to the zwitterionic PC bilayer [14,15], this areal mass may include a layer of water between the bilayer and sensor surface, whose mass has been estimated to be about 102 ng/cm^2^ for PC bilayers supported on silica [45]. Correcting for the mass of this water layer, the mass of the 9:1 PG/LPG bilayer became 343 ng/cm^2^. For the 9:1 PG/LPG mixture, the average molecular weight is 754 g/mol and, correspondingly, the average mass per lipid molecule (M_L_) is 1.25 × 10^−12^ ng/lipid. Dividing the areal mass of the lipid bilayer by the average mass of a single molecule (M_L_), we estimate a surface concentration of the lipid to be 2.74 lipids/nm^2^ in the bilayer and, therefore, the number of lipids per unit area in each leaflet of the bilayer N_L_ is 1.37 lipids/nm^2^ (accounting for the presence of two leaflets constituting the bilayer). This corresponds to a lipid area per molecule of a_L_ = 0.73 nm^2^/lipid. Estimating the molecular volume v_L_ of the hydrophobic tail of the PG and LPG lipids to be 0.96 nm^3^/molecule and 0.46 nm^3^/molecule, respectively, the average v_L_ for the 9:1 PG/LPG mixture is 0.91 nm^3^/molecule (the average volume based on the volumes of the constituent C_16_ and C_18_ chains). Knowing the volume for the molecule and the area per molecule (the molecule refers to one having the average properties of 9:1 PG/LPG), the thickness of the hydrophobic region of the 9:1 PG/LPG bilayer was calculated to be h_L_ = 2 v_L_/a_L_ = 2.49 nm. Using the same procedure, the geometrical properties of the bilayer were calculated for all PG/LPG compositions and the results are reported in Table 3. Also shown for comparison are the values for the zwitterionic Egg PC bilayer estimated in our previous studies [14,15].

The a_L_ and h_L_ values for the anionic PG/LPG lipid mixtures with 10–30% LPG fall in the same range as those estimated [14,15] for the zwitterionic Egg PC bilayer, as shown in Table 4. The estimates for the PG/LPG mixtures are also comparable to the estimates for POPG (16:0–18:1) and DOPG (18:1–18:1) at 30 °C, obtained by the Tristram-Nagle group as a_L_ = 0.66 and 0.71 nm and h_L_ = 2.78 and 2.75 nm, based on neutron scattering and X-ray scattering [46]. In contrast, for the 6:4 PG/LPG, the surface density of lipids is much smaller and the area per lipid a_L_ is significantly larger, indicating that the substrate is not as closely packed with lipids as for other LPG compositions. Since QCM-D provides average values for the film, this larger area per lipid would also be consistent with the existence of lipid patches on the surface with significant gaps between the patches.

The estimated geometrical properties of the bilayers suggest that when the LPG presence in the bilayer exceeds some critical value, it exhibits greater propensity to leave the bilayer during buffer flow. The ability of single-chain lipids, such as LPG, to cause lipid removal from the membrane is a result of their detergent-like nature. Due to the hydrophobicity of the hydrocarbon chains, repulsive interactions between lipid head groups and geometric packing constraints, single-chain amphiphiles tend to form micelles in solution and double-chain amphiphiles will form bilayers, in which lipid head groups are positioned closer together [42]. Adding a large amount of a single-chain lipid into the bilayer changes the average curvature and may promote lipid removal in the form of micelles at some critical composition.

## 4. Conclusions

In this study, we successfully developed a simple method for rapidly forming fully anionic SLBs using various compositions of PG and LPG lipids on APTMS-coated silica. The creation of these stable bilayers was confirmed by monitoring their formation with QCM-D. In the case of Egg PG, a bilayer was not formed but instead we had a supported vesicular layer on the substrate. In contrast, stable bilayers were formed on QCM-D crystals from fully anionic PG/LPG lipid bilayers by using PG/LPG vesicles with 10–30% LPG. The dissipation shifts recorded for these anionic bilayers were similar to that of a rigid PC bilayer. Increasing the LPG concentration to 40% led to lipid removal from the bilayer, likely causing the existence of lipid patches with water-filled gaps on the substrate membrane. The formation of zwitterionic PSC bilayers has been established to be a two-step process of vesicle adsorption followed by vesicle rupture. In contrast, for the PG/LPG mixtures forming a stable bilayer, the process occurs as a single step in which the vesicles reaching the substrate do not adsorb as vesicles, instantaneously rupture and adsorb as bilayers. The area per lipid molecule and the thickness of the hydrophobic region of the bilayer were estimated using the QCM-D data. Although the anionic bilayer thicknesses varied slightly among the different lipid compositions, they were comparable to the properties of the zwitterionic PC bilayer and also of anionic pure POPG and DOPG bilayers. Increased presence of LPG in the mixture at some critical value promotes the loss of lipids from the bilayer, preventing the existence of a continuous bilayer on the quartz crystal surface. These fully anionic membranes are improved models for Gram-positive plasma membranes and will be valuable for examining membrane interactions with other extraneous substances such as antimicrobial peptides or engineered nanoparticles.

## Figures and Tables

**Table 1:** Lipid composition of bacterial inner membranes [29]. Copyright Epand et al, Antimicrob Agents Chemother., 2010.

Bacteria	Lipid Composition (Mole %)		
	PE	PG	CL
**Gram-Positive Bacteria**			
*B*. *polymyxa*	60	3	8
*B*. *cereus*	43	40	17
*E*. *faecalis*	0	27	19
*S*. *epidermis*	0	90	1
*S*. *aureus*	0	57	19
**Gram-Negative Bacteria**			
*E*. *coli*	85	15	5
*K*. *pneumoniae*	82	5	6
*P*. *aeruginosa*	60	21	11

**Table 2:** Current status of formation of anionic lipids * containing SLB for QCM-D.

Lipid Composition	Comments	Ref.
POPC/POPS (75:25)	SLB on silicon oxide-coated quartz crystal; 10 mM MgCl_2_ was added to the buffer for SLB formation. Use of a divalent cation was found to facilitate the SLB incorporating the anionic lipid.	[30]
PE/Egg PC/PS (50:45:5)	SLB on silicon oxide-coated quartz crystal. Acyl chain information for PE and PC not specified in the paper.	[10]
DOPC/DOPS (80:20)	SLB on mica sheet glued to quartz crystal, in the presence of 2 mM CaCl_2_.	[18]
Egg PC/GM1 (98:2, 95:5)	SLB on silicon oxide-coated quartz crystal.	[31]
POPC/Egg PA (80:20) POPC/Egg PG (80:20) POPC/Brain PS (80:20)	SLB on silicon oxide-coated crystal. SLB formation was monitored with or without 3 mM CaCl_2_. POPC/PG with or without Ca^2+^ and POPC/PA without Ca^2+^ form an SLB, while POPC/PS with or without Ca^2+^ and POPC/PA with Ca^2+^ form a layer of unruptured vesicles.	[32]
DMPC/DMPG (80:20)	SLB on gold-coated quartz crystal functionalized with 3-mercaptopropionic acid (MPA) with the carboxyl group of MPA exposed to the vesicles.	[9,33]
Egg PC/*E. coli* IM	SLB on silicon oxide-coated quartz crystal.	[34]
DOPC/DOPS (70:30, 80:20, 90:10) DOPC/DOEPC (70:30, 80:20, 90:10)	SLB on silicon oxide-coated quartz crystal. For the lipid mixture with DOEPC (which is a cationic lipid), vesicle adsorption followed by spontaneous vesicle rupture occurs because of the attractive electrostatic interactions between the positively charged vesicles and the negatively charged crystal surface. It was found that a critical coverage of adsorbed vesicles on the substrate is not necessary to induce spontaneous vesicle rupture.	[19]
DMPC/DMPG (70:30)	SLB on gold-coated quartz crystal functionalized with MPA.	[35]
POPC/DOPIP_3_ (90:10)	SLB on silicon oxide-coated quartz crystal.	[36]
AOT	SLB on silicon oxide-coated quartz crystal at pH of 1.5 (when the crystal surface maintains a positive surface charge).	[37]
DOPC/DOPS (95:5, 90:10, 80:20)	SLB on silicon oxide-coated quartz crystal.	[38]
POPC/POPS (80:20) POPC/POPS/DOPIP_3_ (70:20:10)	Quartz crystal was functionalized with self-assembled monolayers of oligoethyleneglycol with partial ionized carboxyl terminal groups. SLB was formed on this surface in the presence of 2 mM CaCl_2_.	[39]

* Lipid descriptions. POPC (16:0-18:1 PC); POPS (16:0-18:1 PS); DMPC (14:0-14:0 PC); DMPG (14:0-14:0 PG); DOPC (18:1-18:1 PC); DOPS (18:1-18:1 PS); DOPIP_3_ (18:1-18:1 PIP_3_); DOEPC (18:1-18:1 EPC); GM1 (monosialoganglioside); Egg PC is a mixture (16:0 (32.7%), 18:0 (12.3%), 18:1 (32.0%), 18:2 (17.1%)); Egg PA is a mixture (16:0 (34.2%), 18:0 (11.5%), 18:1 (31.5%), 18:2 (18.5%)); Egg PG is a mixture (16:0 (32.9%), 18:0 (12.2%), 18:1 (30.2%), 18:2 (18.7%)); Brain PS is a mixture (18:0 (42%), 18:1 (30%), 22:6 (11%), unknown (15%)); *E. coli* IM (*E. coli* inner membrane isolated from *E. coli*) is a mixture; AOT is a synthetic, anionic surfactant, diethyl hexyl sodium sulfosuccinate, also known as aerosol OT.

**Table 3:** Average slope of frequency shift resulting from vesicle adsorption to the silica surface *.

Vesicle Composition	Average Slope (Hz/min)
PC	−41.4 ± 3.9
PG	−1.4 ± 0.0
9:1 PG/LPG	−13.6 ± 4.2
8:2 PG/LPG	−11.8 ± 1.1
7:3 PG/LPG	−8.8 ± 1.0
6:4 PG/LPG	−12.0 ± 2.5

* Data for adsorption of PG and PG/LPG vesicles on APTMS-coated silica and adsorption of PC vesicles on unfunctionalized silica.

**Table 4:** Calculated values for lipid area per molecule and bilayer hydrophobic region thickness.

Lipid	Avg MW g/mol	Final Δf (Hz)	M_L_ ng/Lipid	N_L_ (Lipids/nm^2^)	a_L_ (nm^2^/Lipid)	v_L_ (nm^3^/Lipid)	h_L_ (nm)
PC	770	−26	1.28 × 10^−12^	1.41	0.71	0.96	2.71
9:1 PG/LPG	754	−25	1.25 × 10^−12^	1.37	0.73	0.91	2.49
8:2 PG/LPG	727	−24	1.21 × 10^−12^	1.35	0.74	0.86	2.32
7:3 PG/LPG	699	−24	1.16 × 10^−12^	1.40	0.71	0.81	2.27
6:4 PG/LPG	672	−18	1.12 × 10^−12^	0.98	1.02	0.76	1.49

## Data Availability

Not applicable.

## Appendix A. Analysis of QCM-D Data

For a rigid film on the crystal surface and when the crystal is in air, the frequency change Δf and the areal mass of the deposited film m_f_ (mass per unit area) are related by the Sauerbrey equation and the dissipation change ΔD, by definition, is zero.

(A1) $$\Delta f=-f_{o}\frac{m_{f}}{m_{q}},\;\Delta D\;=0,\;\Delta m=-C\Delta f.$$

Here, f_o_ is the natural frequency of the oscillator and m_q_ is the areal mass of the quartz crystal. The mass addition (Δm) due to the film deposited on the crystal surface gives rise to a decrease in the frequency (negative Δf) while net mass loss is indicated by a positive Δf. Here, C is a proportionality constant equal to 17.8 ng/cm^2^/Hz for a crystal with a natural frequency of 5 MHz. Note that the use of the Sauerbrey equation is strictly valid for a rigid film on the quartz crystal. The dissipation D is related to the loss modulus G″ and the storage modulus G′ in the form D = G″/(2πG′) and the change in dissipation ΔD is thus related to the viscoelasticity of the film attached to the crystal surface.

For a rigid film on the crystal surface when the crystal is immersed in a Newtonian liquid such as water, the frequency and dissipation changes are modified due to the presence of water and are now given by

(A2) $$\Delta f=-\frac{\eta_{L}}{2{\pi \delta }_{L}m_{q}}-{\;f}_{o}\frac{m_{f}}{m_{q}},\;\Delta D=\frac{\eta_{L}}{{n\pi f}_{o}\delta_{L}m_{q}}$$

where η_L_ is the viscosity of the liquid medium and δ_L_ is the decay length of the acoustic wave in the liquid medium. The first term in the expression for Δf and the only term appearing in ΔD are due to the solvent effect because of the immersion of the crystal in the liquid and they vanish when we consider the changes in the crystal properties after and before the deposition of the rigid film on the crystal surface (since we are measuring difference between two states). Effectively, the film mass changes are given just by the Sauerbrey term and ΔD is zero.

If the film is not rigid but viscoelastic, then the frequency and dissipation changes are given by

(A3) $$\Delta f=-\frac{\eta_{L}}{2{\pi \delta }_{L}m_{q}}-f_{o}\frac{m_{f}}{m_{q}}\;\left[1-\frac{2}{\rho_{f}}{\left(\frac{\eta_{L}}{\delta_{L}}\right)}^{2}\frac{\;G^{\prime \prime }}{{\;G^{\prime }}^{2}+{\;G^{\prime \prime }}^{2}}\right],\;\Delta D\;=+\frac{m_{f}}{m_{q}}\;\left[\frac{4}{\rho_{f}}{\left(\frac{\eta_{L}}{\delta_{L}}\right)}^{2}\frac{\;G^{\prime }}{{\;G^{\prime }}^{2}+{\;G^{\prime \prime }}^{2}}\right]$$

where ρ_f_ is the density of the film on the crystal surface. As in Equation (A2), the first terms in the expressions for Δf and ΔD are due to the solvent effect and they vanish when we consider changes in film properties when the film is immersed in the liquid both before and after the exposure to the vesicle solution. The film mass change is now given by the Sauerbrey term with a correction factor accounting for the viscoelastic properties of the film. To estimate the mass of the film, one will have to simultaneously use the expressions for Δf and ΔD.

There is also a non-vanishing ΔD (the second term in the expression) accompanying the film deposition as shown in Equation (A3). An increase in ΔD corresponds to a decrease in the storage modulus G′ and indicates a less rigid, possibly more disordered film. A decrease in ΔD corresponds to an increase in the storage modulus and indicates a more rigid film on the crystal surface. In experiments involving supported lipid bilayers (SLBs), ΔD can also provide information about changes in the structure and ordering of the lipids. Disruption of the membrane will cause the lipids to become less ordered and potentially allow more water to associate with the membrane, increasing the film’s hydration and ΔD values.

As mentioned already, it is possible to measure not only the changes in the fundamental resonant frequency of the quartz crystal, but also changes in its higher harmonics. Available commercial instruments allow measurements of odd overtones up to 1 the 3th (or even the 15th) multiple of the fundamental frequency. Since higher frequencies dissipate energy faster in a viscous medium, the higher overtones decay faster (the decay length is shorter) and are more confined to the surface region of the crystal. In this study, the 3rd through 11th overtones, or harmonics, were measured. It should be noted that the Δf values are automatically normalized to each overtone so that Δf is the change in f_n_/*n*, where *n* is the harmonic number and f_n_ is the frequency at the nth harmonic. Due to varying penetration depths of the acoustic waves associated with different overtones, higher overtones are qualitatively more representative of processes occurring closer to the crystal–film interface while the lower overtones are representative of processes occurring near the water–film interface.

## References

[1] Clifton L.A., Campbell R.A., Sebastiani F., Campos-Terán J., Gonzalez-Martinez J.F., Björklund S., Sotres J., Cárdenas M. (2020). Design and use of model membranes to study biomolecular interactions using complementary surface-sensitive techniques. Adv. Colloid Interface Sci..

[2] Watts T.H., Brian A.A., Kappler J.W., Marrack P., McConnell H.M. (1984). Antigen presentation by supported planar membranes containing affinity-purified I-Ad. Proc. Natl. Acad. Sci. USA.

[3] McConnell H.M., Watts T.H., Weis R.M., Brian A.A. (1986). Supported planar membranes in studies of cell-cell recognition in the immune system. Biochim. Biophys. Acta.

[4] Richter R.P., Berat R., Brisson A.R. (2006). Formation of solid-supported lipid bilayers: An integrated view. Langmuir.

[5] Keller C.A., Kasemo B. (1998). Surface specific kinetics of lipid vesicle adsorption measured with a quartz crystal microbalance. Biophys. J..

[6] Richter R., Mukhopadhyay A., Brisson A. (2003). Pathways of lipid vesicle deposition on solid surfaces: A combined QCM-D and AFM study. Biophys. J..

[7] Seantier B., Breffa C., Félix O., Decher G. (2005). Dissipation-enhanced quartz crystal microbalance studies on the experimental parameters controlling the formation of supported lipid bilayers. J. Phys. Chem. B.

[8] Hardy G.J., Nayak R., Zauscher S. (2013). Model cell membranes: Techniques to form complex biomimetic supported lipid bilayers via vesicle fusion. Curr. Opin. Colloid Interface Sci..

[9] Mechler A., Praporski S., Atmuri K., Boland M., Separovic F., Martin L.L. (2007). Specific and selective peptide-membrane interactions revealed using quartz crystal microbalance. Biophys. J..

[10] Glasmästar K., Larsson C., Höök F., Kasemo B. (2002). Protein adsorption on supported phospholipid bilayers. J. Colloid Interface Sci..

[11] Rydell G.E., Dahlin A.B., Höök F., Larson G. (2009). QCM-D studies of human norovirus VLPs binding to glycosphingolipids in supported lipid bilayers reveal strain-specific characteristics. Glycobiology.

[12] Kotarek J.A., Moss M.A. (2010). Impact of phospholipid bilayer saturation on amyloid-β protein aggregation intermediate growth: A quartz crystal microbalance analysis. Anal. Biochem..

[13] Wang K.F., Nagarajan R., Mello C.M., Camesano T.A. (2011). Characterization of supported lipid bilayer disruption by chrysophsin-3 using QCM-D. J. Phys. Chem..

[14] Wang K.F., Nagarajan R., Camesano T.A. (2014). Antimicrobial peptide alamethicin insertion into lipid bilayer: A QCM-D exploration. Colloids Surf. B Biointerfaces.

[15] Wang K.F., Nagarajan R., Camesano T.A. (2015). Differentiating antimicrobial peptides interacting with lipid bilayer: Molecular signatures derived from quartz crystal microbalance with dissipation monitoring. Biophys. Chem..

[16] Bailey C.M., Kamaloo E., Waterman K.L., Wang K.F., Nagarajan R., Camesano T.A. (2015). Size dependence of gold nanoparticle interactions with a supported lipid bilayer: A QCM-D study. Biophys. Chem..

[17] Keller C.A., Glasmastar K., Zhdanov V.P., Kasemo B. (2000). Formation of supported membranes from vesicles. Phys. Rev. Lett..

[18] Richter R.P., Brisson A.R. (2005). Following the formation of supported lipid bilayers on mica: A study combining AFM, QCM-D, and Ellipsometry. Biophys. J..

[19] Cho N.-J., Frank C.W., Kasemo B., Höök F. (2010). Quartz crystal microbalance with dissipation monitoring of supported lipid bilayers on various substrates. Nat. Protoc..

[20] Jackman J.A., Choi J.H., Zhdanov V.P., Cho N.J. (2013). Influence of osmotic pressure on adhesion of lipid vesicles to solid supports. Langmuir.

[21] Anderson T.H., Min Y., Weirich K.L., Zeng H., Fygenson D., Israelachvili J.N. (2009). Formation of supported bilayers on silica substrates. Langmuir.

[22] Basit H., Lopez S.G., Keyes T.E. (2014). Fluorescence correlation and lifetime correlation spectroscopy applied to the study of supported lipid bilayer models of the cell membrane. Methods.

[23] Lind T.K., Cárdenas M. (2016). Understanding the formation of supported lipid bilayers via vesicle fusion—A case that exemplifies the need for the complementary method approach. Biointerphases.

[24] Koutsioubas A., Appavou M.S., Lairez D. (2017). Time-resolved neutron reflectivity during supported membrane formation by vesicle fusion. Langmuir.

[25] Lee T.H., Hofferek V., Separovic F., Reid G.E., Aguilar M.-I. (2019). The role of bacterial lipid diversity and membrane properties in modulating antimicrobial peptide activity and drug resistance. Curr. Opin. Chem. Biol..

[26] Harayama T., Riezman H. (2018). Understanding the diversity of membrane lipid composition. Nat. Rev. Mol. Cell Biol..

[27] López-Lara I.M., Geiger O. (2017). Bacterial lipid diversity. Biochim. Biophys. Acta.

[28] Luchini A., Vitiello G. (2021). Mimicking the Mammalian Plasma Membrane: An Overview of Lipid Membrane Models for Biophysical Studies. Biomimetics.

[29] Epand R.F., Pollard J.E., Wright J.O., Savage P.B., Epand R.M. (2010). Depolarization, Bacterial Membrane Composition, and the Antimicrobial Action of Ceragenins. Antimicrob. Agents Chemother..

[30] Travaglia A., Satriano C., Giuffrida M.L., La Mendola D., Rampazzo E., Prodi L., Rizzarelli E. (2013). Electrostatically driven interaction of silica-supported lipid bilayer nanoplatforms and a nerve growth factor-mimicking peptide. Soft Matter.

[31] Weng K.C., Kanter J.L., Robinson W.H., Frank C.W. (2006). Fluid supported lipid bilayers containing monosialoganglioside GM1: A QCM-D and FRAP study. Colloids Surf. B Biointerfaces.

[32] Viitala T., Hautala J.T., Vuorinen J., Wiedmer S.K. (2007). Structure of anionic phospholipid coatings on silica by dissipative quartz crystal microbalance. Langmuir.

[33] Mechler A., Praporski S., Piantavigna S., Heaton S.M., Hall K.N., Aguilar M.I., Martin L.L. (2009). Structure and homogeneity of pseudo-physiological phospholipid bilayers and their deposition characteristics on carboxylic acid terminated self-assembled monolayers. Biomaterials.

[34] Dodd C.E., Johnson B.R.G., Jeuken L.J.C., Bugg T.D.H., Bushby R.J., Evans S.D. (2008). Native *E. coli* inner membrane incorporation in solid-supported lipid bilayer membranes. Biointerphases.

[35] Hasan I.Y., Mechler A. (2016). Formation of planar unilamellar phospholipid membranes on oxidized gold substrate. Biointerphases.

[36] Luchini A., Nzulumike A.N.O., Lind T.K., Nylander T., Barker R., Arleth L., Mortensen K., Cárdenas M. (2019). Towards biomimics of cell membranes: Structural effect of phosphatidylinositol triphosphate (PIP3) on a lipid bilayer. Colloids Surf. B Biointerfaces.

[37] Wolanin J., Barré L., Dalmazzone C., Frot D., Jestin J., Perrot H., Bauer D. (2020). Insight into kinetics and mechanisms of AOT vesicle adsorption on silica in unfavorable conditions. Langmuir.

[38] Feng Y., Zhang Y., Liu G., Liu X., Gao S. (2021). Interaction of graphene oxide with artificial cell membranes: Role of anionic phospholipid and cholesterol in nanoparticle attachment and membrane disruption. Colloids Surf. B Biointerfaces.

[39] John L.H., Preston G.M., Sansom M.S.P., Clifton L.A. (2021). Large scale model lipid membrane movement induced by a cation switch. J. Colloid Interface Sci..

[40] Arouri A., Kiessling V., Tamm L., Dathe M., Blume A. (2011). Morphological changes induced by the action of antimicrobial peptides on supported lipid bilayers. J. Phys. Chem..

[41] Choi E.J., Dimitriadis E.K. (2004). Cytochrome c adsorption to supported, anionic lipid bilayers studied via atomic force microscopy. Biophys. J..

[42] Nagarajan R. (2002). Molecular packing parameter and surfactant self-assembly: The neglected role of the surfactant tail. Langmuir.

[43] Oliver R.C., Lipfert J., Fox D.A., Lo R.H., Doniach S., Columbus J. (2013). Dependence of micelle size and shape on detergent alkyl chain length and head group. PLoS ONE.

[44] Voinova M.V., Jonson M., Kasemo B. (2002). ‘Missing mass’ effect in biosensor’s QCM applications. Biosens. Bioelectron..

[45] Zwang T.J., Fletcher W.R., Lane T.J., Johal M.S. (2010). Quantification of the layer of hydration of a supported lipid bilayer. Langmuir.

[46] Pan J., Heberle F.A., Tristram-Nagle S., Szymanski M., Koepfinger M., Katsaras J., Kučerka N. (2012). Molecular structures of fluid phase phosphatidylglycerol bilayers as determined by small angle neutron and X-ray scattering. Biochim. Biophys. Acta.

